# Development of a Unifying Target and Consensus Indicators for Global Surgical Systems Strengthening: Proposed by the Global Alliance for Surgery, Obstetric, Trauma, and Anaesthesia Care (The G4 Alliance)

**DOI:** 10.1007/s00268-017-4028-1

**Published:** 2017-05-15

**Authors:** Adil Haider, John W. Scott, Colin D. Gause, Mira Meheš, Grace Hsiung, Albulena Prelvukaj, Dana Yanocha, Lauren M. Baumann, Faheem Ahmed, Na’eem Ahmed, Sara Anderson, Herve Angate, Lisa Arfaa, Horacio Asbun, Tigistu Ashengo, Kisembo Asuman, Ruben Ayala, Stephen Bickler, Saul Billingsley, Peter Bird, Matthijs Botman, Marilyn Butler, Jo Buyske, Angelo Capozzi, Kathleen Casey, Charles Clayton, James Cobey, Michael Cotton, Dan Deckelbaum, Miliard Derbew, Catherine deVries, Jeanne Dillner, Max Downham, Natalie Draisin, David Echinard, Sohier Elneil, Ahmed ElSayed, Abigail Estelle, Allen Finley, Erica Frenkel, Philip K. Frykman, Florin Gheorghe, Julian Gore-Booth, Richard Henker, Jaymie Henry, Orion Henry, Laura Hoemeke, David Hoffman, Iko Ibanga, Eric V. Jackson, Pankaj Jani, Walter Johnson, Andrew Jones, Zeina Kassem, Asuman Kisembo, Abbey Kocan, Sanjay Krishnaswami, Robert Lane, Asad Latif, Barbara Levy, Dimitrios Linos, Peter Linz, Louis A. Listwa, Declan Magee, Emmanuel Makasa, Michael L. Marin, Claude Martin, Kelly McQueen, Jamie Morgan, Richard Moser, Robert Neighbor, William M. Novick, Stephen Ogendo, Akinyinka Omigbodun, Bisola Onajin-Obembe, Neil Parsan, Beverly K. Philip, Raymond Price, Shahnawaz Rasheed, Marjorie Ratel, Cheri Reynolds, Steven M. Roser, Jackie Rowles, Lubna Samad, John Sampson, Harshadkumar Sanghvi, Marchelle L. Sellers, David Sigalet, Bruce C. Steffes, Erin Stieber, Mamta Swaroop, John Tarpley, Asha Varghese, Julie Varughese, Richard Wagner, Benjamin Warf, Neil Wetzig, Susan Williamson, Joshua Wood, Anne Zeidan, Lewis Zirkle, Brendan Allen, Fizan Abdullah

**Affiliations:** 1 Department of Surgery, Brigham and Women’s Hospital, Boston, MA USA; 2 Center for Surgery and Public Health, Brigham and Women’s Hospital, Boston, MA USA; 3 Division of Pediatric Surgery, Department of Surgery, Ann & Robert H. Lurie Children’s Hospital of Chicago, 225 East Chicago Ave, Box 63, Chicago, IL 60611 USA; 4 The Global Alliance for Surgical, Obstetric, Trauma, and Anaesthesia Care, New York, NY USA; 5 Selfless, London, UK; 6 ReSurge International, Sunnyvale, CA USA; 7 The Pan African Association of Surgeons, Parktown, Johannesburg, South Africa; 8 Department of Surgery, Mayo Clinic Florida, Jacksonville, FL USA; 9 Society of American Gastrointestinal and Endoscopic Surgeons, Los Angeles, CA USA; 10 St. Paul Medical College, Addis Ababa, Ethiopia; 11 Jhpiego, An Affiliate of Johns Hopkins University Baltimore, Baltimore, MD USA; 12 African Agency for Integrated Development, Kampala, Uganda; 13 Operation Smile, Virginia Beach, VA USA; 14 Alliance for Surgery and Anaesthesia Presence, Lupsingen, Switzerland; 15 FIA Foundation, London, UK; 16 AIC Kijabe Hospital, Kijabe County, Kenya; 17 Netherlands Society for International Surgery, Amsterdam, The Netherlands; 18 Global Pediatric Surgery Network, Portland, OR USA; 19 American Board of Surgery, Philadelphia, PA USA; 20 Rotaplast International, San Francisco, CA USA; 21 Primary Trauma Care Foundation, Oxford, UK; 22 Johns Hopkins School of Public Health, Baltimore, MD USA; 23 International Collaboration for Essential Surgery, Angwin, CA USA; 24 Centre for Global Surgery, Montreal, QC Canada; 25 McGill University Health Centre, Montreal, QC Canada; 26 The College of Surgeons of East, Central and Southern Africa, Arusha, Tanzania; 27 University of Utah Center for Global Surgery, Salt Lake City, UT USA; 28 SIGN Fracture International, Richland, WA USA; 29 International College of Surgeons, Chicago, IL USA; 30 HumaniTerra, Marseille, France; 31 Fistula Foundation, San Jose, CA USA; 32 Alzaiem Alazhari University, Khartoum North, Sudan; 33 Willing and Abel, Norfolk, VA USA; 34 ChildKind International, Boston, MA USA; 35 Gradian Health Systems, Inc, New York, NY USA; 36 Global Pediatric Surgical Technology and Education Project, Irvine, CA USA; 37 Arbutus Medical Inc, Vancouver, Canada; 38 World Federation of Societies of Anaesthesiologists, London, UK; 39 American Association of Nurse Anesthetists, Park Ridge, IL USA; 40 Henry Family Advised Fund, Chicago, IL USA; 41 IntraHealth International, Chapel Hill, NC USA; 42 Healing the Children, Orlando, FL USA; 43 Pro-Health International, Edwardsville, IL USA; 44 Christiana Care Health System, Newark, DE USA; 45 WHO Global Initiative for Emergency and Essential Surgical Care, Geneva, Switzerland; 46 Tropical Health and Education Trust, London, UK; 47 Roads for Life, Beirut, Lebanon; 48 Kupona Foundation, Saratoga Springs, NY USA; 49 World Journal of Surgery, Portland, OR USA; 50 Oregon Health and Science University, Portland, OR USA; 51 International Federation of Surgical Colleges, Bogis-Bossey, Switzerland; 52 Department of Anesthesiology and Critical Care Medicine, Johns Hopkins University, Baltimore, MD USA; 53 American College of Obstetricians and Gynecologists, Washington, DC USA; 54 Institute of Preventive Medicine, Environmental and Occupational Health - Prolepsis, Attica, Greece; 55 National and Kapodistrian University, Athens Medical School, Athens, Greece; 56 Mercy Ships, Lindale, TX USA; 57 Kenya Society of Anaesthesiologists, Nairobi, Kenya; 58 Royal College of Surgeons of Ireland, Dublin, Ireland; 59 Permanent Mission of the Republic of Zambia to the United Nations, Geneva, Switzerland; 60 Mount Sinai Health System, New York, NY USA; 61 AO Alliance Foundation, Davos, Switzerland; 62 Vanderbilt University Medical Center, Nashville, TN USA; 63 Physicians for Peace, Norfolk, VA USA; 64 Solidarity Bridge, Evanston, IL USA; 65 Diamedica UK Ltd, Devon, UK; 66 University of Tennessee Health Science Center, Memphis, TN USA; 67 William Novick Global Cardiac Alliance, Memphis, TN USA; 68 West African College of Surgeons, Lagos, Nigeria; 69 Nigerian Society of Anesthetists, Lagos, Nigeria; 70 Organization of American States, Washington, DC USA; 71 American Society of Anesthesiologists, Schaumburg, IL USA; 72 The Institute of Global Health Innovation, Imperial College London, London, UK; 73 Korle-Bu Neuroscience Foundation, Langley, BC Canada; 74 Assist International, Scotts Valley, CA USA; 75 International Association of Oral and Maxillofacial Surgeons, Chicago, IL USA; 76 International Federation of Nurse Anesthetists, Sursee, Switzerland; 77 Indus Hospital Pakistan, Karachi, Pakistan; 78 Global Surgery Initiative, Johns Hopkins University, Baltimore, MD USA; 79 Mending Kids, Burbank, CA USA; 80 World Federation of Associations of Pediatric Surgeons, Geneva, Switzerland; 81 Pan African Academy of Christian Surgeons, Linden, NC USA; 82 Smile Train, New York, NY USA; 83 Association for Academic Surgery, Los Angeles, CA USA; 84 GE Foundation, Chicago, IL USA; 85 AmeriCares, Stamford, CT USA; 86 Global ENT Outreach, Coupeville, WA USA; 87 CURE International, Lemoyne, PA USA; 88 HEAL Africa, Gisengyi, Rwanda; 89 Plasticos Foundation, Newport Beach, CA USA; 90 IVUmed, Salt Lake City, UT USA; 91 2nd Chance Association Reconstructive Surgery for Life Reconstruction, Meyrin, Switzerland

## Abstract

After decades on the margins of primary health care, surgical and anaesthesia care is gaining increasing priority within the global development arena. The 2015 publications of the Disease Control Priorities third edition on *Essential Surgery* and the Lancet Commission on Global Surgery created a compelling evidenced-based argument for the fundamental role of surgery and anaesthesia within cost-effective health systems strengthening global strategy. The launch of the *Global Alliance for Surgical, Obstetric, Trauma, and Anaesthesia Care* in 2015 has further coordinated efforts to build priority for surgical care and anaesthesia. These combined efforts culminated in the approval of a World Health Assembly resolution recognizing the role of surgical care and anaesthesia as part of universal health coverage. Momentum gained from these milestones highlights the need to identify consensus goals, targets and indicators to guide policy implementation and track progress at the national level. Through an open consultative process that incorporated input from stakeholders from around the globe, a global target calling for safe surgical and anaesthesia care for 80% of the world by 2030 was proposed. In order to achieve this target, we also propose 15 consensus indicators that build on existing surgical systems metrics and expand the ability to prioritize surgical systems strengthening around the world.

## Introduction

Surgical care, encompassing surgery, obstetrics, trauma, and anaesthesia, is needed to address nearly one-third of the global burden of disease [1]. This need remains unmet for billions of people, as less than 6% of all surgeries are performed in the world’s poorest countries, despite representing more than two-thirds of the world’s population [2]. The majority of individuals in these low- and middle-income countries (LMIC) do not have access to essential surgical care, with an estimated 143 million additional surgical procedures needed annually to bridge the gap [2]. People in resource-limited settings continue to suffer due to a lack of trained healthcare providers, inadequate health system infrastructure, disproportionate out-of-pocket healthcare costs, and a lack of prioritization of surgical care as part of national health plans [2, 3]. With the recent approval of World Health Assembly (WHA) resolution 68.15 recognizing the importance of emergency and essential surgical care and anaesthesia as part of universal health coverage [4], and the introduction of the United Nations (UN) Post-2015 Sustainable Development Goals (SDGs) [5, 6], there is a critical need for consensus regarding strategies to support advocacy, resource mobilization, and strengthening of surgical systems as part of national healthcare plans worldwide.

The past 2 years have witnessed unprecedented engagement by academic, public health, government and multilateral organizations in advocating for the inclusion of safe surgical and anaesthesia care as part of the global health and policy agenda. Recent milestones include the publication of the World Bank Disease Control Priorities 3rd Edition (DCP3) Volume on *Essential Surgery* [7], and the Lancet Commission on Global Surgery (LCoGS) report that demonstrated a lack of access to surgery for 5 billion people worldwide [2]. In May 2015, the Global Alliance for Surgical, Obstetric, Trauma, and Anaesthesia Care (G4 Alliance) was officially launched as a coalition of 20 organizations dedicated to providing a voice for the billions of neglected surgical patients around the world [8]. Building on the pioneering efforts of numerous other groups, including the World Health Organization’s (WHO) Global Initiative for Emergency and Essential Surgical Care (GIEESC), the G4 Alliance serves to support WHO efforts and others by providing a much needed advocacy platform for the prioritization of surgical care as part of the global development agenda.

The DCP3 provides evidence on intervention efficacy and cost-effectiveness for the leading causes of the current global disease burden. The inclusion of a volume on essential surgery helped to define the avertable burden of surgically treatable disease and the potential impact of increasing access to surgical care to reduce the overall global disease burden. The LCoGS engaged individuals from over 110 countries to produce an evidence base and a compelling argument for investment in surgical care. In May 2015, the combined efforts of numerous organizations and stakeholders culminated in the unanimous approval by 194 WHO Member States of a WHA resolution dedicated to addressing the importance of surgical and anaesthesia care [4]. Resolution WHA68.15, “Strengthening emergency and essential surgical care and anaesthesia as a component of universal health coverage”, issues a call to countries to adopt and implement policies that support prioritization and integration of safe, high-quality, and cost-effective surgical care and anaesthesia as part of existing health systems [4, 9, 10].

In only 24 months, the G4 Alliance has grown to become a network of over 80 organizations around the world, including non-profit organizations, academic institutions, professional societies, federations, and private sector partners. The Alliance seeks to build political will and public health prioritization of surgical, obstetric, trauma, and anaesthesia care in support of WHA 68.15 as part of the global development agenda [4]. The G4 Alliance advances this goal through advocacy, policy implementation, and resource mobilization efforts. Its membership is purposefully diverse, with dues-paying member organizations in over 140 countries. Member organizations include experts in general surgery, obstetrics, trauma, anaesthesia, and other surgical specialties, as well as nursing, midwifery, and other non-physician surgical providers, disparate disciplines that have historically worked independent of one another yet are vital to strengthening surgical care. This manuscript describes the process and outcomes of a consensus building process that was used to develop a unifying target and consensus indicators for Global Surgical Systems Strengthening that has been approved by this very diverse alliance.

## Global consultation process

**Fig. 1:**
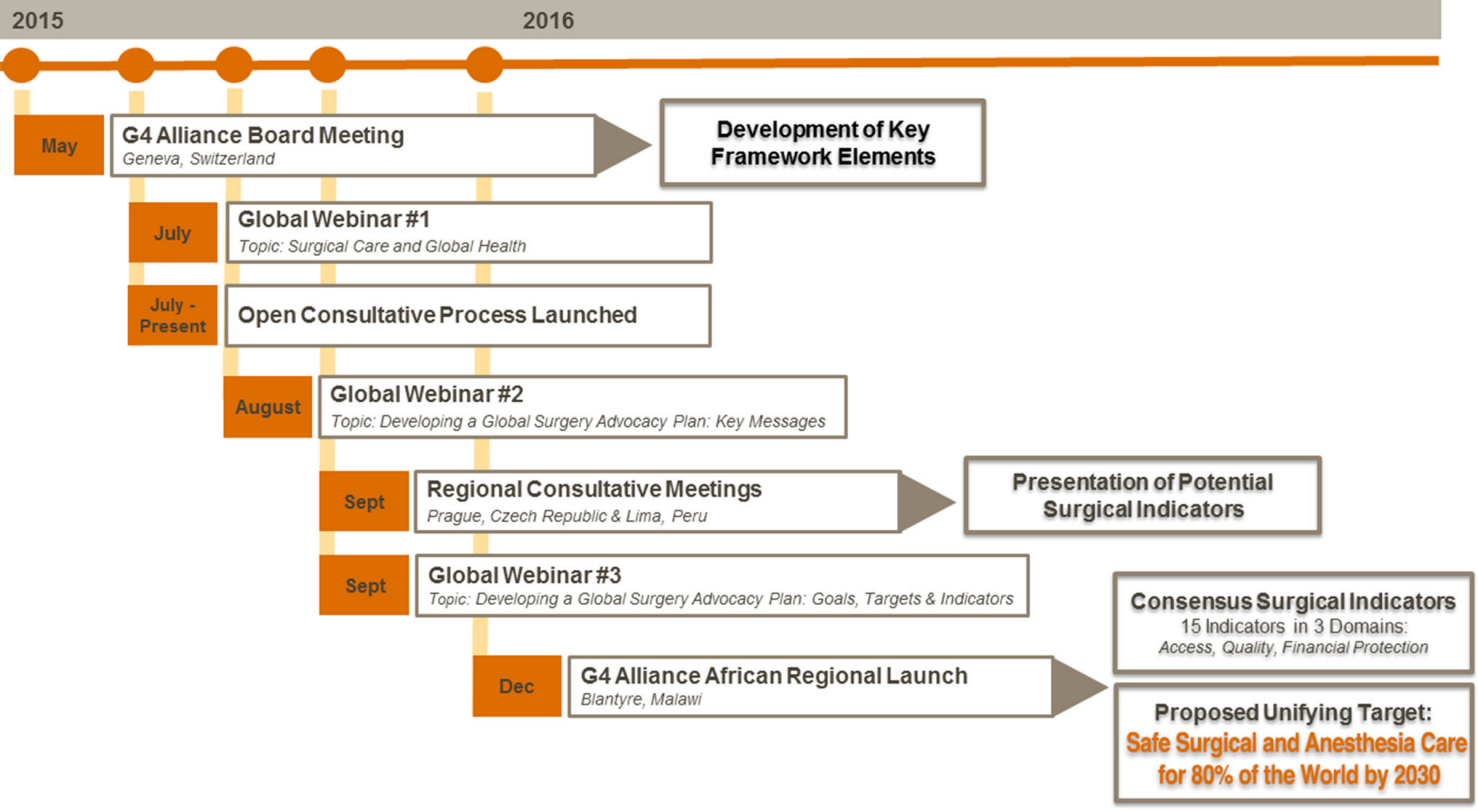
Timeline of the global consultative process for the development of surgical indicators and the unifying target for safe surgical and anaesthesia care

The G4 Alliance implemented a global consultation process (Fig. 1) that includes the engagement of expert working groups to create consensus recommendations regarding global surgical care. While this consultative process is ongoing, recommendations have already emerged, including (1) the importance of developing a framework of multidisciplinary and cross-cutting metrics that reflect the critical role of surgical, obstetric, trauma, and anaesthesia care services at the first-referral level, and (2) the need for coordinated global targets to provide specific, time-bound and actionable objectives that will guide the response of global stakeholders. Similar strategies have been successfully employed by other global health movements [11].

## Development of the G4 alliance platform

### Development of surgical indicators and global targets

Key outputs from the G4 Alliance’s consultative process include cross-disciplinary goals, targets, and indicators as well as a framework of international standards and guidelines that can be adapted to local and regional contexts. This consultative process began with the establishment of an expert working group on goals, targets, and indicators. This group first defined the primary purpose of surgical indicators: (1) to serve as tools for advocacy, quality, and patient-centred care at the local, national, and international level; (2) to guide decision making around surgical services at the local and national level; and (3) to assist fundraising and resource mobilization efforts by demonstrating existing needs and goal-oriented progress over time.

In addition, the working group established that all indicators considered in the process must: (1) build and strengthen existing initiatives, especially building upon established priority indicators put forth by the LCoGS [2], the WHO’s Core 100 List of Health Indicators [12], the World Bank’s World Development Indicators (WDIs) [13], and the UN SDGs [5]; (2) be practical and feasible to collect; (3) be applicable at the international, national, and regional levels; (4) contribute to the G4 Alliance’s aim to collectively represent diverse socioeconomic and multidisciplinary stakeholders invested in improving surgical care; and (5) support a unifying target to help align global efforts.

Establishing consensus surgical indicators began with a detailed review of existing repositories of health indicators, including the LCoGS [2], WHO’s Core 100 List of Health Indicators [12], World Bank’s WDIs [13], UN SDGs [5], and indicators utilized in other global health advocacy efforts [11]. During this review, it was noted that these groups had already done exceptional work. In particular, the six indicators proposed by the LCoGS for surgical systems strengthening were extremely relevant and timely. It was additionally felt that there was a need to add indicators that also synergistically represented obstetric, trauma, and anaesthesia care along with those for surgery in order to create a more nuanced picture of a health system’s surgical and anaesthesia care capacity. Thus, the working group began with the six core surgical indicators established by the LCoGS [2] and then incorporated additional metrics that reflect the synergistic role of providing surgical, obstetric, trauma, and anaesthesia care. As part of this process, the G4 Alliance hosted three global webinars throughout 2015, bringing together multidisciplinary content experts, member organizations, and members of the general public. Additionally, specific input was sought from expert stakeholders from across disciplines of surgical, obstetric, trauma, and anaesthesia care.

Initial recommendations were consolidated by the working group and presented during G4 Alliance regional consultative meetings held in Prague, Czech Republic, during the International College of Surgeons Jubilee World Congress, and during the Confederation of Latin American Societies of Anaesthesiologists Congress (CLASA; the World Federation of Societies of Anesthesiologists’ Latin American Regional Section) in Lima, Peru, both in September 2015. Emerging ideas were introduced and debated by attendees during these regional meetings, helping the working group to achieve a consensus. A summary of the consultative process is included in Fig. 1.

A preliminary set of consensus indicators and a proposed unifying global target were introduced at the G4 Alliance’s Board Meeting, African Regional Launch Event and Consultation held in Blantyre, Malawi, in December 2015. During these meetings, proposed consensus indicators were introduced, debated, and refined in breakout sessions and plenaries. After a period of public reporting and open discussion, the list of 15 consensus surgical indicators for global surgical, obstetric, trauma, and anaesthesia care (Table 1) was established, along with a global unifying target. This list is comprised of the six core surgical indicators from the recent LCoGS [2], as well as nine other multidisciplinary surgical indicators previously established by either the WHO or other professional associations [12, 14–17]. The multidisciplinary framework of the 15 consensus indicators includes three key domains: access, quality, and financial risk protection. While each indicator provides unique information, they are purposefully interdependent and designed to be implemented and monitored as part of a complete surgical care package.

**Table 1:** Proposed indicators to monitor and evaluate surgical systems

Domain	Best for	Indicator	Reference
Access	Surgical system	Access to timely essential surgery^†^	WHO Core 100**
		Specialist surgical workforce density^†^	WHO Core 100**
	Trauma care	Estimated proportion of seriously injured patients transported by ambulance	WHO IMR
	Trauma and obstetrics	National whole blood donation rate	WHO GDBS
	Obstetrics	C-section rate	WHO Core 100+
	Anaesthesia	Proportion of operating theatres with pulse oximetry	WHO PSPOP
		Ratio of anaesthetists to surgeons	WHO Core 100**
Quality	Surgical system	Surgical Volume^†^	WHO Core 100**
		Perioperative mortality rate (POMR)^†^	WHO Core 100
	Trauma care	Inpatient trauma mortality rate	ACS COT
	Obstetrics	Maternal Mortality Ratio (proportion due to maternal haemorrhage, obstructed labour)	WHO Core 100**
		Neonatal mortality	WHO Core 100
	Anaesthesia	POMR on operative day	WHO Core 100**
Financial risk protection	Surgical system	Protection against impoverishing expenditure^†^	WHO Core 100**
		Protection against catastrophic expenditure^†^	WHO Core 100**

^†^Core LCoGS measure for surgical systems strengthening, WHO Core 100: Worth Health Organization’s Global Reference List of 100 Core Health Indicators, 2015, WHO Core 100** the surgically relevant indicator can be disaggregated from existing Core 100 indicators, WHO Core 100+ signifies a Core 100 “Additional Indicator”, WHO IMR: WHO’s Indicator and Measurement Registry, WHO GBDS: WHO’s Global Database on Blood Safety, WHO PSPOP: WHO’s Patient Safety Pulse Oximetry Project, ACS COT: American College of Surgeons Committee on Trauma

### Consensus surgical care indicators

Throughout this consultative process, there was significant discussion regarding the need to balance the adoption of key indicators of surgical care with the burden of implementation and national-level data collection. Collecting these surgical indicators requires a dedicated workforce and a process by which governments can receive sustainable support for the collection and analysis of data. This process requires manpower and resources, representing potential hurdles to tracking and reporting. Fortunately, many of these indicators are currently being collected, decreasing the burden on national health systems.

#### Access to surgical care

The first two indicators include access to timely essential surgery and specialist surgical workforce density. In accordance with the LCoGS and the WHO Core 100, timely access is defined as the proportion of the population that live within 2-h travel time to a facility that can provide services including caesarean section, laparotomy, long bone fracture repair and cranial surgery, while surgical workforce density is defined as the number of trained surgeons, anaesthetists, and obstetricians per 100,000 population [2, 12]. Taken together, these two population-level indicators provide an overall measure of the availability of a basic level of surgical care.

The estimated proportion of seriously injured patients transported by ambulance and the caesarean section (C-section) rate provide a more complete understanding of access to trauma and obstetric care [12, 14]. Previously used as a proxy for timely access to trauma care [18], the proportion of injured patients transported by ambulance is an important access measure which has been established as a “pillar indicator” by the UN’s Road Safety Collaboration and the WHO [19]. In the 2015 Statement on Caesarean Section Rates [20], the WHO affirms the live-saving role of C-sections, when indicated. While rates above 10% have not been linked to reductions in maternal and newborn mortality rates [20], tracking the C-section rates is critical to identify both insufficient access and overuse. Critical for all surgical patients, the WHO report on blood safety highlights the “major imbalance between developing and developed countries in the level of access to safe blood” with a median national whole blood donation rate of 36.4 donations/1000 population in HICs versus 2.8 in LICs [21]. As such, national whole blood donation rate provides an important proxy for both resource availability and systems development [15].

Anaesthesia is essential to safe and pain-free surgery and represents a major contributor to positive outcomes after any intervention [22]. Access to safe anaesthesia care underpins any surgical care delivery system and can be monitored by tracking the ratio of anaesthetist to surgeon and the proportion of operating theatres with pulse oximetry [16]. Tracking and improving the ratio of anaesthetist to surgeon is necessary to address the critical lack of trained anaesthesia providers globally [23]. Additionally, the proportion of operating theatres with pulse oximetry, previously established as a marker for the availability of essential surgical equipment [24], is critical to providing safe surgical and anaesthesia care. Notably, up to 70% of district hospitals in sub-Saharan Africa do not have pulse oximetry available [25], further underscoring the importance of implementing this measure. These proxy measures correlate with access in terms of both human and material resources and provide a more detailed description of the distribution and availability of safe anaesthesia.

#### Surgical care quality

The G4 Alliance joins others in promoting perioperative mortality rate (POMR) as a fundamental quality metric for surgery [2, 12]. Defined as death on the day of surgery, death before discharge, or death within 30 days of procedures, POMR is a feasible, credible, and critical measure of surgical quality [2, 13, 25, 26]. As case mix and volume are integrally related to mortality risk, POMR should be considered in the context of overall surgical volume, measured in cases per 100,000 population [2, 12]. While day of surgery mortality may be secondary to a myriad of causes including severity of patient’s illness, case complexity, intraoperative decision making, and resource availability, it may also reflect the quality of anaesthesia care. Documenting the presence of untreated head injury as part of the multi-trauma patient population will inform the POMR in the presence of unavailable timely imaging and surgical resources. Areas without the capacity to provide high-quality, safer anaesthesia care will have a much higher perioperative mortality rate within 24 h of surgery. Additionally, day of surgery POMR will provide more much meaningful information regarding the safety of anaesthesia care than will 30-day POMR. As such, longitudinal tracking of day of surgery POMR can provide important insight into the safety of anaesthesia care [2, 12, 22]. Although these measures may be coarse, they serve as a requisite starting point for any surgical system seeking to improve the quality of care delivery.

The current standard for assessing quality of trauma care is the inpatient trauma mortality rate [17, 27]. When viewed alongside the trauma-related access measure of proportion of injured patients receiving ambulance transport, these two indicators provide an overall sense of both access to and quality of trauma care. Similarly, the quality of obstetric care is determined by not only access to caesarean section, but by the maternal mortality ratio (MMR), with specific attention paid to the proportion of MMR related to surgically amenable causes such as maternal haemorrhage and obstructed labour [12]. Often used to interpret the benefit of population-level access to C-sections [28], Neonatal mortality rate^1^ represents another important indicator related to timely access to safe surgical care. Taken together, these two indicators are crucial to the development of a surgical system that can significantly impact both maternal and child health care.

#### Financial risk protection

Findings from the LCoGS suggest that approximately 33 million people worldwide face catastrophic health expenditures each year, directly attributable to costs related to surgical services [2]. When factoring in non-medical costs, this estimate increases to 81 million people [2]. Simply put, there is no quality without access, and there is no access if millions of individuals cannot afford essential surgical services. As such, the G4 Alliance promotes the use of indicators to measure protection against catastrophic expenditure^2^ as well as protection against impoverishing expenditure [2, 12].^3^ These measures are calculated as the proportion of households that are protected from either catastrophic or impoverishing expenditures due to direct out-of-pocket costs of surgical care.

#### Monitoring surgical indicators

The proposed surgical indicators (Table 1) rely on established indicators that can serve as a proxy for systems capacity rather than disease-specific clinical data. We recommend that health systems conduct routine and standardized collection of data to allow comparisons over time and between locations. Routine assessments of burden further allow for advocacy around prioritization of specialty care as a part of the overall surgical community, with indicators specific to a disease or condition (Table 2). Such assessments will allow national health organizations to focus on areas of need within the broader field of surgical care itself, including specialty disciplines.

**Table 2:** Proposed indicators to monitor sub-specialty care

Burden	Population-level incidence and prevalence measures
	DALYs attributed to condition; proportion of DALYs avertable by treatment
Access	Proportion of population able to access facilities providing condition-specific care
	Sub-specialist providers per 100 K population
Quality	Annual volume of sub-specialty procedures
	Post-operative mortality/morbidity
Financial protection	Inclusion into national insurance coverage
	Protection against impoverishing and catastrophic expenditure

### Unifying global target


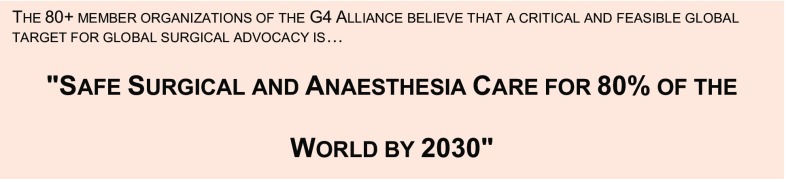
The indicator framework is designed to measure the ability of a health system to provide safe, high-quality, accessible surgical care. Demonstrating sustained progress across these interdependent indicators is critical to achieve universal access to high-quality surgical, obstetric, trauma, and anaesthesia care. After consensus consideration, the G4 Alliance established its unifying global target as “Safe Surgical and Anaesthesia Care for 80% of the World by 2030”. This global target is meant to be applied at the global, national, and subnational level, incorporating the interdependent nature of access, quality, and universal health coverage. As up to 5 billion people currently lack access to safe surgical care [2], urgent action is required worldwide to meet the critical needs of the neglected surgical patient.

## The way forward

The G4 Alliance and its member organizations are committed to achieving the global goal of “safe surgical and anaesthesia care for 80% of the world by 2030”. The proposed 15 consensus indicators were chosen so that we can critically monitor and evaluate efforts, as we collectively create a path towards this goal. These consensus indicators integrate the collective interests of surgery, obstetrics, trauma, and anaesthesia by providing interdependent measures of access, quality, and financial risk protection. They have been purposefully drawn from existing indicators, which will facilitate monitoring and evaluating ongoing surgical systems strengthening efforts. Ultimately, their collective adoption will be critical to effective implementation of safe surgical systems for the world’s neglected surgical patients.

Immediate action is required to translate academic, political, and advocacy efforts into tangible, effective implementation of surgical care delivery. As an initial step, countries are encouraged to develop national surgical plans (NSPs) to assess their own strengths, weaknesses, needs, and goals regarding safe and effective surgical care delivery. It is reassuring to note that in response to the LCoGS nearly a dozen countries are already beginning to develop NSPs, which is a very welcome occurrence. Many of these countries are being assisted by the LCoGS and are focusing their initial indicator data collection on the six core LCoGS indicators for surgical systems. In addition, the World Bank is considering inclusion of these core measures in their World Development Indicators, which will make their collection even more sustainable and durable. As an alliance, we applaud these initial efforts and believe that collection of these six core indicators will lay the groundwork for the collection and utilization of the broader 15 consensus indicators presented in Table 1. (The six core indicators mentioned above are included within these 15 consensus indicators.) Indicators are critical components to guide both the development and the monitoring of progress within a national surgical plan. As these plans begin to emerge, collection and monitoring of indicators may vary based on individual country circumstances. For reasons of sustainability and feasibility, many may choose to begin data collection with a more limited set such as the six core surgical indicators proposed by LGoGS. This is understandable, and we agree that at a minimum, all NSPs should include collection of these measures. However, as an alliance we strongly recommend countries to quickly develop the capability to gather and utilize all 15 of the consensus indicators presented in this document so that a more complete understanding of a nations’ Surgery, Anaesthesia, Trauma, and Anaesthesia care can be ascertained and responded to. Continued collaboration throughout the surgical community is needed to establish best practices to support development of NSPs for countries in critical need of surgical systems strengthening.

Moving forward, consensus indicators must be incorporated into a new global accountability framework for surgical care, which will allow clinicians, governments, funders, local and international organizations to unite around common goals and targets, and track progress over time. This framework is needed to expand upon the key messages championed by the DCP3, the LCoGS, WHO GIEESC, and the G4 Alliance [2, 7, 8] and promote international standards and guidelines to evaluate health systems improvements. An agile data platform is also needed to help avoid siloed efforts and improve economies of scale through interconnectivity and alignment of goals and stakeholders, while tracking developments in clinician training standards as well as ongoing partnerships.

Building consensus for indicators and global targets represents an essential step towards developing a global accountability framework for surgical care, as a critical component of health, human rights, and economic growth. Revisiting the call to action shared by Dr. Hafdan Mahler, WHO Director General in 1980, these efforts are important to ensure that “surgery will play its proper role in bringing the people of the world nearer to the goal of health for all….” [29] Surely by achieving safe surgical and anaesthesia care for 80% of the world by 2030, we will be closer to achieving this goal for humanity.

## Footnotes

1 Number of deaths before 28 days of age per 1000 births.

2 Expense that exceeds 40% of annual post-subsistence income.

3 Expense that causes a household to fall below the poverty line.
